# Efficacy of the 7-chloro-4-(3-hydroxy-benzilidenehydrazo) quinoline derivative against infection caused by *Leishmania amazonensis*

**DOI:** 10.1590/0037-8682-0091-2020

**Published:** 2020-06-22

**Authors:** Luciana Maria Ribeiro Antinarelli, Marcus Vinicius Nora de Souza, Eduardo Antonio Ferraz Coelho, Wallace Pacienza Lima, Elaine Soares Coimbra

**Affiliations:** 1 Universidade Federal de Juiz de Fora, Campus Universitário, Departamento de Parasitologia, Microbiologia e Imunologia, Juiz de Fora, MG, Brasil.; 2 Universidade Federal de Minas Gerais, Faculdade de Medicina, Programa de Pós-Graduação em Ciências da Saúde: Infectologia e Medicina Tropical, Belo Horizonte, MG, Brasil.; 3 Fundação Oswaldo Cruz, Instituto de Tecnologia em Fármacos-FarManguinhos, Rio de Janeiro, RJ, Brasil.; 4 Universidade Federal de Minas Gerais, Colégio Técnico, Departamento de Patologia Clínica, Belo Horizonte, MG, Brasil.; 5 Universidade Federal do Rio de Janeiro, Instituto de Biofísica Carlos Chagas Filho, Laboratório de Imunofarmacologia, Rio de Janeiro, RJ, Brasil.; 6 Universidade do Grande Rio, Escola de Ciências da Saúde, Duque de Caxias, RJ, Brasil.

**Keywords:** Leishmania amazonensis, Quinoline, Chemotherapy, Cutaneous leishmaniasis, Toxicity

## Abstract

**INTRODUCTION:**

The drugs currently available for leishmaniasis treatment have major limitations.

**METHODS:**

*In vitro* and *in vivo* studies were performed to evaluate the effect of a quinoline derivative, Hydraqui (7-chloro-4-(3-hydroxy-benzilidenehydrazo)quinoline, against *Leishmania amazonensis*. *In silico* analyses of absorption, distribution, metabolism, excretion, and toxicity (ADMET) parameters were performed.

**RESULTS:**

Hydraqui showed significant *in vitro* anti-amastigote activity. Also, Hydraqui-treated mice exhibited high efficacy in lesion size (48.3%) and parasitic load (93.8%) reduction, did not cause hepatic and renal toxicity, and showed appropriate ADMET properties.

**CONCLUSIONS:**

Hydraqui presents a set of satisfactory criteria for its application as an antileishmanial agent.

Leishmaniasis is a parasitic infection caused by over 20 species of protozoa of the genus *Leishmania.* It is characterized by distinct clinical presentation and is dependent on the tropism of the infecting species fort issues such as the skin, mucous membranes, or visceral organs^1^. An estimated 14 million people have been infected across 98 countries^2^. Cutaneous leishmaniasis (CL) presents a high incidence, with an estimated 1-1.5 million new cases occurring worldwide each year. The most recent report estimated 0.3 million new cases of visceral leishmaniasis, the most lethal form of the disease, resulting in over 20,000 deaths each year^1^. 

Depending on the *Leishmania* species and the host immune response, CL manifests with distinct clinical and pathological features^2^. Localized cutaneous leishmaniasis is the most prevalent clinical form, characterized by the presence of single or multiple ulcerating lesions, which appear at the site of inoculation by the insect vector^2^. Mucosal leishmaniasis has a more severe manifestation marked by the development of disfiguring lesions in mucosal membranes of the naso-oropharyngeal cavity and is clinically characterized by an intense inflammatory response, tissue damage, and low parasite load^2^ ^,^ ^3^. Diffuse cutaneous leishmaniasis, a non-ulcerative form of the disease, is accompanied by an immune response triggered by damaged cells, intense parasitism in the lesions, and poor response to chemotherapy^2^ ^,^ ^3^.

Currently, the most effective drugs used to treat leishmaniasis are pentavalent antimonial-derived products. Amphotericin B deoxycholate and its liposomal formulations, pentamidine and paromomycin, are recommended as alternative drugs. However, all available therapeutic options present limitations in terms of long treatment time, intravenous route, systemic toxicity, high cost, therapeutic failure, disease relapse, and/or emerging parasite resistance^4^ ^,^ ^5^. Miltefosine is still the only approved oral therapeutic option; however, frequent relapse cases, risk of drug-resistant parasites, and varying cure rates against CL in Central and South American countries have led to severe restrictions on the application of this therapy^5^.

Quinoline derivatives have distinct pharmacological applications, including the treatment of bacterial, viral, and protozoa infections. Moreover, it exhibits anti-inflammatory activity and can be used to treat cancer^6^. Several studies have explored this class of scaffolds as promising alternatives for the development of new and selective antileishmanial agents, and some molecules have been identified^7^ ^,^ ^8^. Consequently, the current study involving this quinoline derivative class of compounds represents a promising strategy to identify new and potent antileishmanial compounds. 

In the previous study, we demonstrated the promising *in vitro* antileishmanial effects of a series of derivatives of 7-chloro-4-quinolinyl and hydrazine groups^9^. Among the evaluated compounds, the majority had low toxicity against macrophages and showed significant activity against the promastigotes of four *Leishmania* species and intra-macrophage amastigotes of *L. braziliensis* (IC_50_<1 µM). Continuing our investigation with this series of compounds, in this study, we evaluated the *in vitro* and *in vivo* effect of 7-chloro-4-(3-hydroxy-benzilidenehydrazo)quinoline derivative, Hydraqui, the most effective molecule identified, against *L. amazonensis* infection for the first time. 

For *in vitro* analysis of anti-amastigote activity, peritoneal macrophages were allowed to adhere to coverslips placed in 24-well culture plates for 16 h. Then, *L. amazonensis* (IFLA/BR/67/PH8) stationary promastigotes were added to the wells, and the cultures were incubated for 4 h at 33 °C in 5% CO_2_. The infected macrophages were treated with Hydraqui (5-100 µM) for 72 h at 33 ºC in 5% CO_2_ followed by Giemsa staining. The results were evaluated by counting the intracellular amastigotes present in the infected macrophages. IC_50_ values were obtained of three independent experiments by using the Probit program. Hydraqui was previously synthesized by our research group and characterized using spectroscopic and spectrometric methods^9^. Amphotericin B (AmpB; Cristália, SP, Brazil; 0.06-1.0 µM) was used as a reference drug. 

For the *in vivo* assay, BALB/c mice (female, 8-10 weeks of age, 20-25 g of body weight) were obtained from the Biology Research Center at the Federal University of Juiz of Fora (CEUA protocols ≠056/2013 and ≠046/2014). Mice were infected with 2 × 10^6^ *L. amazonensis*-GFP stationary-phase promastigotes. After 7 days of infection, mice were divided into three groups (5 mice per group): (i) Control group: treated with 10 µL of 1× PBS containing 5% DMSO; (ii) AmpB group: treated with 10 μL of 1× PBS containing 250 µg/Kg AmpB; and (iii) Hydraqui group: treated with 10 μL of 1× PBS containing 100 µg/Kg Hydraqui. AmpB and Hydraqui were diluted in a solution of 1× PBS containing 5% DMSO. Mice were treated twice a week and a total of eight doses were administered subcutaneously at the site of infection. Lesion development was monitored before each treatment using a dial caliper (Digimess, SP, Brazil)^7^. To evaluate the treatment efficacy, mice were euthanized three days after the final treatment (35 days post-infection) and the number of viable parasites in the ears of each mouse was estimated using the limiting dilution assay^7^. The parasite burden was measured using fluorometry, as previously described^8^.

For systemic toxicity analysis, sera samples were collected at the end of the experiment, and the levels of alanine aminotransferase (ALT), aspartate transaminase (AST), gamma-glutamyl transpeptidase (GGT), and creatinine were analyzed using laboratory colorimetric kits (Labtest Diagnostica®, Belo Horizonte, Minas Gerais, Brazil)^7^. In addition, the weight of each animal was monitored before, during, and after the treatment.

For *in silico* prediction analysis of drug-likeness, the theoretical properties of absorption, distribution, metabolism, excretion, and toxicity (ADMET) of Hydraqui were evaluated using the admetSAR tool, and the Lipinski’s rule of five was calculated using the Molsoft *in silico* software^10^. 

Data were statistically analyzed using the One-way-ANOVA followed by Dunnett’s test to compare the treated groups to the negative controls using the Prism 5 software (GraphPad Software, San Diego, CA, USA).

The present study was the first of its kind to demonstrate the *in vitro* and *in vivo* activity of a 7-chloroquinoline derivative, Hydraqui, against *L. amazonensis*, a species which presents a significant clinical challenge due to its diverse manifestations during CL, and in some cases, of visceral leishmaniasis in humans, as well its frequent association with poor response to or failure of treatment^2^.

The results of the current study showed that Hydraqui displays significant *in vitro* activity during treatment of infected macrophages (Figure1A and B). Evaluation of the degree of infection showed that the infection rates were 58.37%, 50.33%, 61.87%, 32.28%, and 16.81% (Figure 1A), and the number of recovered amastigotes were 3.82, 3.28, 3.25, 1.06, and 0.46 (Figure 1B) following treatment with 5, 10, 25, 50, and 100 μM of Hydraqui, respectively. The negative control (infected, but untreated macrophages) showed an infection rate of 79.1%, while the number of amastigotes was 6.07. Based on these results, the IC_50_ value for Hydraqui was 7.32 µM, whereas the IC_50_ for AmpB was 0.07 µM. 

**FIGURE 1:**
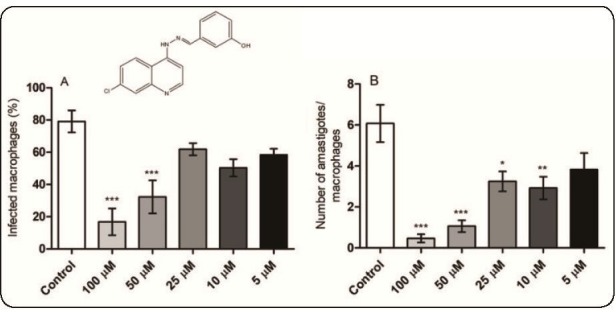
**Effect of Hydraqui in the treatment of *L. amazonensis*-infected macrophages.** Murine macrophages were infected with *L. amazonensis* and treated with the indicated concentrations of **Hydraqui** for 72 h at 33 ºC/5% CO_2_. After the cells were stained with Giemsa, the percentage of infection **(A)** and the number of recovered amastigotes **(B)** were calculated by counting 200 cells, in duplicate. Data were expressed as the means of three independent experiments, which were performed in duplicate. ***P<0.001; **P<0.01, and *P<0.1 (compared with control group).

In this study, the subcutaneous route of treatment of *L. amazonensis*-infected mice at the infection site was chosen to investigate the *in vivo* therapeutic effects of Hydraqui. The World Health Organization recommends the inclusion of intralesional treatment among the acceptable therapeutic alternatives, mainly in countries in Central and South America, where the spontaneous cure rate is relatively low for CL^11^. Local treatment of CL offers several advantages over systemic therapy, including the use of lower drug doses, easy administration, improved safety, non-invasive routes, better patient compliance, fewer adverse effects, and cost-effectiveness^11^.

The *in vivo* therapeutic action of Hydraqui was evaluated by measuring the lesion size and estimating the parasitic load in the infected ears of mice (Figure 2A-D). Our results showed that the progression of lesions was significantly lower after administration of 100 µg/kg of Hydraqui compared to that in the control group (Figure 2A). Lesion growth was significantly reduced (48.3%) in the Hydraqui group compared with the control group (Figure 2B). Treatment with AmpB (at the highest concentration tested) also proved to be effective in controlling lesion growth. Quantification of the parasitic load using the limiting dilution assay showed that the parasitic load was 93.8% lower in the experimental group treated with Hydraqui than in the control group (Figure 2C). Indirect quantification of the parasitic burden using fluorometry revealed that the parasitic load was 65.0% lower in the experimental group treated with Hydraqui than in the control group (Figure 2D). Significant reduction in the parasitic load was also observed in the AmpB group (Figure 2C and 2D). These findings are in agreement with the results of other studies in the literature, which demonstrated that the subcutaneous route of administration is safe and can achieve the antileishmanial effect of 7-chloroquinoline derivatives *in vivo* ^7^ ^,^ ^8^. 

**FIGURE 2:**
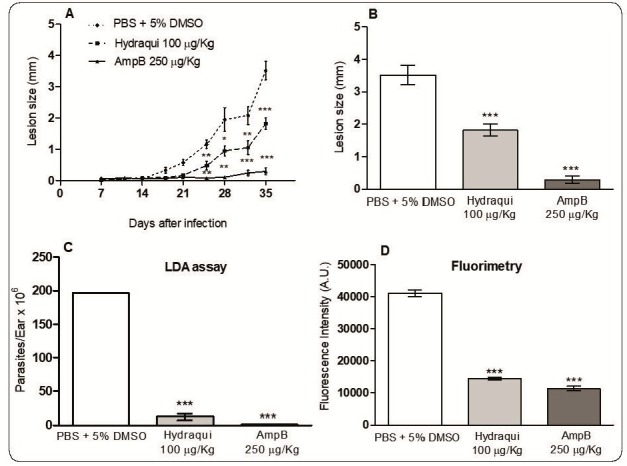
**Efficacy of Hydraqui in the treatment of *L. amazonensis-*infected mice.** The effect of **Hydraqui** treatment on lesion size was evaluated by measuring the lesion with a dial caliper twice a week until the end of the treatment **(A)**. Lesion size at the end of the treatment (35 days post-infection) **(B)**. Evaluation of the parasite load in the infected tissues 35 days post-infection using limiting dilution analysis (LDA) **(C),** and by fluorometric assay **(D)**. Fluorometric values are expressed as specific fluorescence units (FU) and are corrected for background uninfected ear values. ***P<0.001, **P<0.01, and *P<0.1 (compared with control group).

It is interesting to note that Hydraqui did not show any antileishmanial activity against the promastigotes of *L. amazonensis* ^9^. However, in the present study, Hydraqui was effective *in vitro* against the intracellular forms (IC_50_ ≤ 10 μM) and showed significant *in vivo* reductions in lesion size and parasitic load. Stage-specific variations in activities have been widely reported for *Leishmania* species *in vitro* and *in vivo*, but the reasons are not well understood. Several factors may contribute to the *in vitro* and *in vivo* variation in sensitivity, including differences in pH, physicochemical profiles favoring entry of the drug into cells via diffusion across the infected macrophages, and drug metabolism, among others^12^ ^,^ ^13^. Pentavalent antimony is a classic example, as this antileishmanial drug is more effective against the amastigotes than promastigotes of *Leishmania* species in *in vitro* assays and also exhibits strong effects *in vivo* ^14^.

Analysis of the *in vivo* systemic toxicity of Hydraqui in the infected and treated animals was performed at the end of the treatment by measuring the levels of the serum markers of hepatic damage (AST, ALT, and GGT) and renal toxicity (creatinine). The Hydraqui treatment regimen did not affect these markers, indicating the safety of the treatment via the subcutaneous route. Analysis of the experimental group treated with AmpB revealed an increase in ALT and creatinine levels (Figure 3A and 3D). No significant change in body weight was observed in groups treated with Hydraqui or AmpB compared with the control group (data not shown).

**FIGURE 3:**
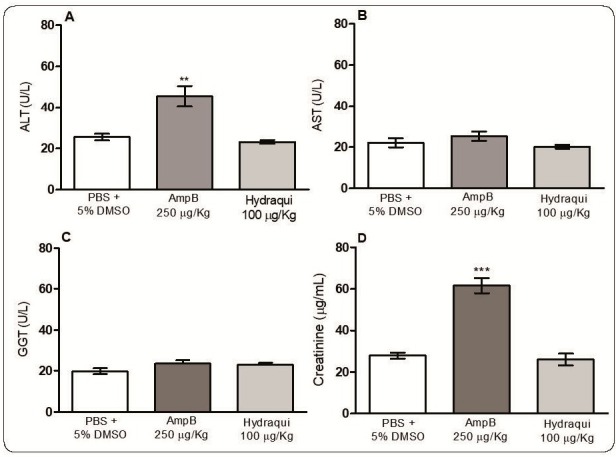
Evaluation of systemic toxicity in the treated mice. After 72 h of the end of the treatment period, serum samples were collected for the determination of the levels of (A) alanine aminotransferase (ALT), (B) aspartate transaminase (AST), (C) gamma-glutamyl transpeptidase (GGT) and (D) creatinine, using colorimetric diagnostic kits. Results are expressed as mean ± standard deviation, and ***P<0.001 and **P<0.01 represent significant difference compared to the control group.

Although the Hydraqui treatment regimen in this study involved the subcutaneous route of administration, theoretical analysis of the physicochemical characteristics (Lipinsky's rule) and ADMET properties were performed to evaluate the safety and bioavailability of this compound following oral administration for future application^15^. Results revealed that Hydraqui presents optimum physicochemical properties, including low molecular weight (297.15), moderate lipophilicity (LogP = 5.15), and few functional groups for hydrogen bonding propensity (including four hydrogen-bond acceptors and two donors, data not shown). The calculated ADMET properties showed that Hydraqui has a high probability of human intestinal absorption and Caco-2 cell permeability (96.71% and 55.08%, respectively), suggesting a good profile of oral bioavailability. Regarding its toxicity, this study found that Hydraqui has a class III risk of acute toxicity (as for compounds with median lethal dose [LD_50_] > 500 mg/kg^11^). A drawback, however, is that Hydraqui demonstrates a high probability of inhibiting the enzymatic activities of CYP450 1A2 and CYP450 3A4, the cytochrome P450 enzymes responsible for drug metabolism (data not shown). These results highlight the potential for administration of Hydraqui through the oral route.

In conclusion, our results indicate that Hydraqui presents a set of satisfactory criteria for its application as an antileishmanial agent. It was highly effective in the treatment of intra-macrophage amastigotes (IC_50_ value ≤ 10 μM) and demonstrated low toxicity in murine macrophages. Hydraqui-treated mice showed a significant reduction in lesion size and parasitic load, which correlated with the low levels of toxicity markers and appropriate ADMET properties. These findings warrant further studies on Hydraqui to improve its therapeutic efficacy in the treatment of *Leishmania* spp infections.
